# Osteodystrophy in Cholestatic Liver Diseases Is Attenuated by Anti-γ-Glutamyl Transpeptidase Antibody

**DOI:** 10.1371/journal.pone.0139620

**Published:** 2015-09-29

**Authors:** Yusuke Kawazoe, Mutsumi Miyauchi, Atsuhiro Nagasaki, Hisako Furusho, Syunryo Yanagisawa, Chea Chanbora, Toshihiro Inubushi, Hideyuki Hyogo, Takashi Nakamoto, Keiko Suzuki, Sawako Moriwaki, Susumu Tazuma, Shumpei Niida, Takashi Takata

**Affiliations:** 1 Department of Oral and Maxillofacial Pathobiology, Institute of Biomedical and Health Sciences, Hiroshima University, Hiroshima, Japan; 2 Department of Medicine and Molecular Science, Institute of Biomedical and Health Sciences, Hiroshima University, Hiroshima, Japan; 3 Department of Oral and Maxillofacial Radiology, Institute of Biomedical and Health Sciences, Hiroshima University, Hiroshima, Japan; 4 Department of Pharmacology, School of Dentistry, Showa University, Tokyo, Japan; 5 Biobank Omics Unit, National Center for Geriatrics and Gerontology, Aichi, Japan; 6 Departments of General Internal Medicine, Hiroshima University Hospital, Hiroshima, Japan; Texas A&M Health Science Center, UNITED STATES

## Abstract

**Background:**

Cholestatic liver diseases exhibit higher levels of serum γ-glutamyl transpeptidase (GGT) and incidence of secondary osteoporosis. GGT has been identified as a novel bone-resorbing factor that stimulates osteoclast formation. The aim of this study was to elucidate the interaction of elevated GGT levels and cholestatic liver disease-induced bone loss.

**Methods:**

Wistar rats were divided into three groups: sham-operated control (SO) rats, bile duct ligation (BDL) rats, and anti-GGT antibody-treated BDL rats (AGT). Serum GGT level was measured. Bone mineral density (BMD) was analyzed by dual-energy X-ray absorptiometry. Bone morphometric parameters and microarchitectural properties were determined by micro-computed tomography and histomorphometry of the distal metaphysis of femurs. Alterations of bone metabolism-related factors were evaluated by cytokine array. Effects of GGT on osteoblasts or stromal cells were evaluated by RT-PCR, enzyme activity, and mineralization ability.

**Results:**

Serum levels of GGT were significantly elevated in the BDL-group. In the BDL group, BMD, bone mass percentage, and osteoblast number were significantly decreased, whereas osteoclast number was significantly increased. These alterations were markedly attenuated in the AGT group. The mRNA levels of vascular endothelial growth factor-A, LPS-induced CXC chemokine, monocyte chemoattractant protein-1, tumor necrosis factor-α interleukin-1β and receptor activator of nuclear factor-kappa B ligand were upregulated, and those of interferon-γ and osteoprotegerin were downregulated in the GGT-treated stromal cells. Furthermore, GGT inhibited mineral nodule formation and expression of alkaline phosphatase and bone sialo-protein in osteoblastic cells.

**Conclusion:**

Our results indicate that elevated GGT level is involved in hepatic osteodystrophy through secretion of bone resorbing factor from GGT-stimulated osteoblasts/bone marrow stromal cells. In addition, GGT also possesses suppressive effects on bone formation. Managing elevated GGT levels by anti-GGT antibody may become a novel therapeutic agent for hepatic osteodystrophy in chronic liver diseases.

## Introduction

Osteoporosis and osteomalacia are the most common complications in patients with chronic liver diseases and cholestatic liver diseases (CLD) [1–7]. Since these patients are always at a higher risk of fractures not associated with trauma [6], timely treatment is needed to diminish the risk of fractures and maintain a satisfactory quality of life. The causal mechanisms of osteoporosis induced by CLD appear to be diverse and multifactorial. Indeed many factors including genetic aberrations, abnormal calcium, vitamin D, vitamin K, and bilirubin levels, and alcohol consumption have been reported. However, underlying mechanisms have not been fully elucidated [1,2,6].

γ-glutamyl transpeptidase (GGT) is a type II transmembrane protein synthesized in epithelial cells lining the intrahepatic bile duct, and serves as a key enzyme in the catabolism of glutathione (GSH) and cysteine metabolism [8]. Serum levels of GGT are significantly upregulated in hepatic diseases including CLD, and have been used as a useful biomarker of liver damage. On the other hand, Niida and his colleagues, using an expression cloning strategy, identified GGT as a novel bone-resorbing factor that activated osteoclast formation independently of enzymatic activity [9]. They further demonstrated that overexpression of GGT induced not only upregulation of serum GGT but also severe bone destruction in vivo [10]. Using bile duct ligation (BDL) rat as a model of CDL, we showed that elevated GGT levels in CLD serum are implicated in decreased bone mass, and anti-GGT antibody treatment attenuated the CLD-induced bone loss.

## Materials and Methods

### Animals and reagents

Seven-week-old male Wistar rats weighing 200–250 g (N = 30) were purchased from Charles River Japan (Shizuoka, Japan), Recombinant human GGT (rhGGT) was produced in *Spodoptera frugiperda* Sf2 cells using a baculovirus system.[11] Monoclonal antibody against rhGGT (AGT3), which can neutralize the osteoclast forming activity of GGT, was kindly provided by AC-Biotechnology (Yokohama, Japan).

### Animal treatment and bile duct ligation rat model

This study was carried out in strict accordance with the recommendations in the Guide for the Care and Use of Laboratory Animals of the Hiroshima University Animal Research Committee and AVMA Guidelines on Euthanasia. The protocol described below was approved by the Committee on the Ethics of Animal Experiments of the Hiroshima University (Permit Number: A11-43). All mice were housed in a specific pathogen free facility in 12 hr light-dark cycles with access to water and food ad libitum. Rats were closely monitored by the body weights and general health.

During the operation, all experimental Rats were anaesthetized by intraperitoneal injection of Somnopentyl (54mg/Kg; Kyouritu Seiyaku, Tokyo, Japan) and atropine sulfate (416μg/Kg; Mitsubishi Tanabe Pharma Co. Osaka, Japan) and all efforts were made to minimize suffering.

Animals were divided into three groups: bile duct ligation (BDL) group, AGT3-treated BDL (AGT) group and the control sham-operated (SO) group. In the BDL and AGT groups, the common bile duct was exposed and double ligated with 6–0 silk sutures, and then the peritoneum and skin were closed; the AGT group was treated with intraperitoneal administration of AGT3 (50 μg/100g/day) for 2 days from before BDL to the end of the experiment. The rats in the SO group underwent a median laparotomy with isolation of the common bile duct without ligation.

Animals were euthanized 2 weeks after BDL by ethyl ether, and blood was collected from the inferior vena cava along with femora were also collected. Serum GGT, aspartate transaminase (AST), alanine aminotransferase (ALT), total bilirubin, and 1,25-dihydroxyvitamin D3 (1,25(OH)_2_D_3_) levels were measured by SRL Inc. (Tokyo, Japan). The weight of animals was recorded at the time of surgery and at time of sacrifice.

### Bone analysis

**Fig 1:**
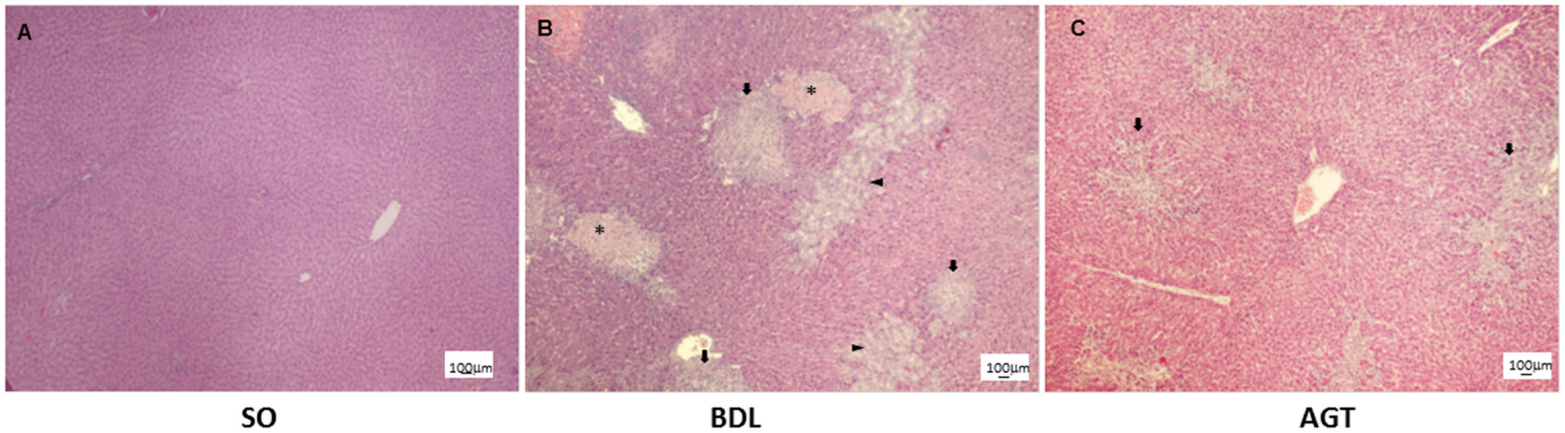
Bile duct ligation caused liver injury such as necrosis, fibrosis and bile duct proliferation and AGT3 partially improves them. Histological findings of liver at 2 w after operation. SO, sham operated, control rats; BDL, rats with cholestatic liver disease treated by bile duct ligation; and AGT, BDL rats treated with intraperitoneal injection of GGT antibody (AGT3). Asterisk; necrotic area, arrow; fibrosis area and arrow head; bile duct proliferation area. H&E. Scare bars are 100μm.

Bone mineral density assessment was performed on the lower body of the rat (white square in Fig 1A) using dual-energy X-ray absorptiometry (DEXA, DPX-alpha small animal total body mode; Lunar, Madison, WI, USA). The femora of the rats (N = 3) were fixed for 1 day in 10% buffered formalin, and then stored in 70% ethanol. Bone microarchitecture properties of femora were evaluated by microcomputed tomography (μ-CT) using an inspeXio SMX-90CTTM (Shimadzu Co., Kyoto, Japan) with X-ray tube settings of 90 kV and 108 μA. A total of 280 projections were scanned at a voxel size of 15.0 μm. TRI/3D-BONTM software (RATOC System Engineering Co., Tokyo, Japan) was used to generate three-dimensional models and perform bone morphometry in the distal end of each femur.

### Histological analysis

The left femora were fixed in periodic-lysine paraformaldehyde solution at 4°C for 24 h and decalcified in 1 mM phosphate-buffered saline (PBS; pH 7.4) containing 10% EDTA for 10 days at 4°C and subsequently embedded in paraffin; serial sections (4.5 μm thick) were cut and mounted on glass slides. The sections were then stained with hematoxylin-eosin (H&E) and Azan for histological examination. Some sections were stained for tartrate-resistant acid phosphatase (TRAP) activity to detect osteoclasts according to the method of Minkin.[12] The H&E stained specimens obtained from the liver tissues of each experimental group were also made.

For histomorphometric analysis, the right femora were fixed in 70% ethanol and embedded in glycolmethacrylate without decalcification (N = 6). Sections were stained with Villanueva bone stain and analyzed with bone histomorphometry software at the Ito Bone Science Institute (Niigata, Japan).

### Cytokine array

Rat Cytokine Antibody Array G series 2 was purchased from Ray-Biotech Inc. (Norcross, GA). A volume of 50 μL of bone marrow flushed out from the long bone was collected and centrifuged at 10,000 rpm for 1 min (4°C). The supernatants were analyzed according to the manufacturer’s instructions. The arrays were quantified using GenePix (Molecular Devices, California) fluorescent scanner and the data analyzed using Gene Pix Pro 6.0 and RayBiotech rat cytokine G series software.

### Osteoclastogenesis

#### Cell culture

The bone marrow-derived osteogenic cell line ST2 cells and primary osteoblasts (OBs) obtained from newborn mice (C57BL/6) calvariae were maintained in Minimum Essential Medium Alpha medium (α-MEM) (Invitrogen Corp., N.Y. U.S.A.) with 10 mM HEPES (pH 7.2), 10% fetal bovine serum (FBS) (Invitrogen Corp.) and 100 U/ml penicillin-streptomycin (Invitrogen Corp.) at 37°C in a humidified atmosphere of 5% CO_2_.

### Gene expression experiments

ST2 cells were seeded in 35-mm culture dishes (1 × 10^6^ cells/dish) and incubated in α-MEM containing 10% FBS for 2 d. After changing medium, the cells were further incubated with rhGGT (100 ng/ml), and harvested before and 2, 4, 12, and 24 h after rhGGT stimulation. Another set of cell cultures were pretreated with or without AGT3 (100 ng/ml) for 2 h before rhGGT addition, and then cells were harvested 2 h after rhGGT stimulation. Total RNA was extracted from the harvested cells using TRIzol® reagent (Invitrogen Corp.). cDNA was synthesized from 1 μg of total RNA using the ReverTra Dash (TOYOBO Co., LTD., Osaka, Japan) kit. Aliquots of total cDNA were amplified using KOD-Plus-DNA Polymerase (TOYOBO Co.). Amplification was performed using a MyCycler^TM^ thermal cycler (BIO-RAD, Tokyo, Japan). PCR products were electrophoresed on 1.5% agarose gels at 100 mV and visualized by ethidium bromide staining. The primer pairs used in the present study are listed in Table 1. The primer pair and probe for 18S are commercially available (Applied Biosystems, CA, USA).

**Table 1:** PCR primers and probe used in this study.

Gene	Forward	Revers
Mouse		
ALP	5’-TCCCTACCGACCCTGTTGTGA-3’	5’-TGGACCTCTCCCTTGAGTGT -3’
BSP	5’-GTACCGGCCACGCTACTTTCT-3’	5’-GTTGACCGCCAGCTCGTTTT-3’
TNF-α	5'-ATGAGCACAGAAAGCATGATC-3'	5'-TACAGGCTTGTCACTCGAATT-3'
IL-1β	5’-AGAGAGCCTGTCTTTTCCTCCTTG-3’	5’-GCTTCAATGAAAGACCTCAGTGCAG-3’
VEGF-A	5'-CCT CCG AAA CCA TGA ACT TTC TGC TC-3'	5'-CAG CCT GGC TCA CCG CCT TGG CTT-3'
LIX	5'-GAAAGCTAAGCGGAATGCAC-3'	5'-GGGACAATGGTTTCCCTTTT-3'
MCP-1	5'-AGGTCCCTGTCATGCTTCTG-3'	5'-TCTGGACCCATTCCTTCTTG-3'
IFN-γ	5'-TTTGAGGTCAACAACCCACA-3'	5'-CGCAATCACAGTCTTGGCTA-3'
RANKL	5'-CGCTCTGTTCCTGTACTTTCGAGCG-3'	5'-TCGTGCTCCCTCCTTTCATCAGGTT-3'
OPG	5'-CAGAGACTAATAGATCAAAGGCAGG-3'	5'-ATGAAGTCTCACCTGAGAAGAACC-3'
18S	5’-CGCTATCTGACTCGCTG-3’	5’-GGAAGGTTCTAGTCAGG-3’

Quantitative real time PT-PCR was performed in the Applied Biosystem StepOnePlus™ (Appleid Biosystems), using TaqMan® Fast Advanced Master Mix (Appleid Biosystems) and specific primers and probe for TNF-α (Forward: CCAAATGGCCTCCCTCTCAT, Reverse; GCTACAGGCTTGTCACTCGAATT, Probe; 5’-FAM-CCCAGACCCTCACACACTCAGATCATCTTC-TAMRA-3’) and MCP-1 (Forward: GCTGGAGAGCTACAAGAGGATCAC, Reverse; TGGTTCCGATCCAGGTTTTTA Probe; 5’-FAM-CAGCAGGTGTCCCAAAGAAGCTGTAGTTTTT-TAMRA-3’). Reaction product was quantified with 18S (X03205.1 Eukaryotic 18S rRNA; Appleid Biosystems) as the reference gene.

### Western blotting

The cells were pretreated with or without AGT3 (100 ng/ml) for 2 h followed by 100ng/ml of rhGGT, and then cells were harvested 1day or 2 d after rhGGT stimulation. Western blotting was carried out as previous description [9]. Cell pellets were resuspended in ice-cold lysis buffer as described previously. Proteins were separated by SDS-PAGE and electro-blotted onto nitrocellulose membrane. The membrane was blocked by 3% milk for 30min. Pro-IL-1β was detected by polyclonal IL-1β antibody (1:500; Santa Cruz Biothechnology, Inc. Texas, USA) and anti-rabbit secondary antibody. Monoclonal anti-β-actin (1:10000, Sigma-Aldrich Co., MI, USA) was used as internal control. The results were visualized by Amersham ECL western blotting detection system (GE Healthcare, Japan).

### Osteoclast differentiation *in vitro*

Bone marrow cells (BMCs) were isolated from C57BL mice and plated in 10cm dish for 2 d. 5x10^4^ of non-adherent BMCs were distributed into 96 well-plate and incubated in the presence of 30ng/ml of mouse M-CSF (R&D system, MN, USA) for 2 d. The conditioned medium of ST2 cells stimulated for 2 d with 1μg/ml of hrGGT was added to adherent BMCs for 5 d. To investigate the effect of rhGGT induced TNF-α, neutralizing antibody of TNF-α (ab9635: abcam, MA, USA) was applied simultaneously with rhGGT. The medium was refreshed every 2 d. TRAP staining was performed to detect osteoclasts.

### Osteogenic differentiation

#### Alkaline phosphatase activity

Primary OBs were plated into 24 well plates (3×10^3^ cells/well) and cultured in the medium described above. After 4 d, the medium was changed and cells were treated with 10, 100, and 500 ng/ml of rhGGT. At 7 d after treatment with rhGGT, alkaline phosphatase (ALP) activity was quantitatively analyzed by the Bessey-Lowry enzymology method [13] (Alkaline-phospha B test; Wako, Osaka, Japan).

#### Gene Expression Experiments

Cells were plated in 35-mm culture dishes (1× 10^6^ cells/dish) containing MEM medium. Then medium was changed to α-MEM containing 10% FBS, amino acids (50 μg/ml), and sodium β-glycerophosphate (10 mM) with rhGGT (10, 100, 500 ng/ml). At 7 d after rhGGT-stimulation, the cultured cells were harvested and the expression of mineralization-related genes was analyzed. The primer pairs used are also listed in Table 1.

#### Mineralization

Primary OBs were plated in 24 well plates (3 × 10^4^ cells/well) and cultured in α-MEM supplemented with 10% FBS. Then medium was changed to fresh α-MEM containing 10% FBS, ascorbic acid (50 μg/ml), and sodium β−glycerophosphate (10 mM) with rhGGT (0, 10, 100 and500 ng/ml). After 3 w, the cells were fixed in a 3.5% formaldehyde neutral buffered solution, and stained with alizarin red S (ALZ) to confirm the calcified nodules. Next, to evaluate the amount of ALZ-stained cells per dish, the stained cell-cultures were further incubated with 5 ml 0.1 mol/L cetylpyridinium chloride, and absorbance was measured at 570 nm. The concentrations were calculated according to the standard curve.

### Statistical analysis

Data are presented as mean ± (SD). Statistical differences among experimental groups were evaluated by One-way ANOVA followed by Tukey’s post-test using SSRI for windows (Social Survey Research Information Co., Ltd., Tokyo, Japan) with the level of significance described for p < 0.01 (**) and p < 0.05 (*).

## Results

### Body weight, serum biomarkers and liver condition

Body weights and serum biochemical marker levels were measured (Table 2). The final body weight in the BDL group was significantly lower than that of the SO group (*P* = 0.0002). Total bilirubin (S1 Table), GGT, AST, and ALT levels in the BDL group were significantly higher than those in the SO group (*P* < 0.0001). By contrast, active metabolite Vit.D3 (1,25(OH)_2_D_3_) levels were significantly downregulated (*P* < 0.0001; S1 Table). Moreover, AGT application significantly reduced serum levels of biomarkers like GGT, AST and ALT indicating the improvement of liver injury. Therefore we histologically examined liver tissue of each group. Surprisingly, AGT application partially improved BDL-induced necrosis, fibrosis and bile duct proliferation (Fig 1).

**Table 2:** Body weight and biochemical parameters.

	SO	BDL	AGT
Initial body weight (g)	285.40 ± 5.42	282.00 ± 3.62	283.20 ± 4.64
Final body weight (g)	332.10 ± 5.57	308.20 ± 5.79^a^	310.80 ± 5.31^a^
Serum GGT (IU/l)	0.33 ± 0.49	40.67 ± 9.18^a^	11.20 ± 6.09^a^ ^,^ ^b^
Serum AST (IU/l)	140.40 ± 28.95	542.30 ± 145.62^a^	357.90 ± 118.02^a^ ^,^ ^b^
Serum ALT (IU/l)	37.50 ± 8.41	134.60 ± 23.18^a^	89.60 ± 36.57^a^ ^,^ ^b^

SO, sham-operated group; BDL, bile duct ligated group; AGT3, AntiGGTantibody treated group, GGT, gammer glutamyl transpeptidase; IU, international unit; AST, aspartate transaminase; ALT, alanine aminotransferase.

^a^: p<0.01 vs SO,

^b^: p<0.01 vs BDL

### Bone morphometry of bile duct ligation animals

**Fig 2:**
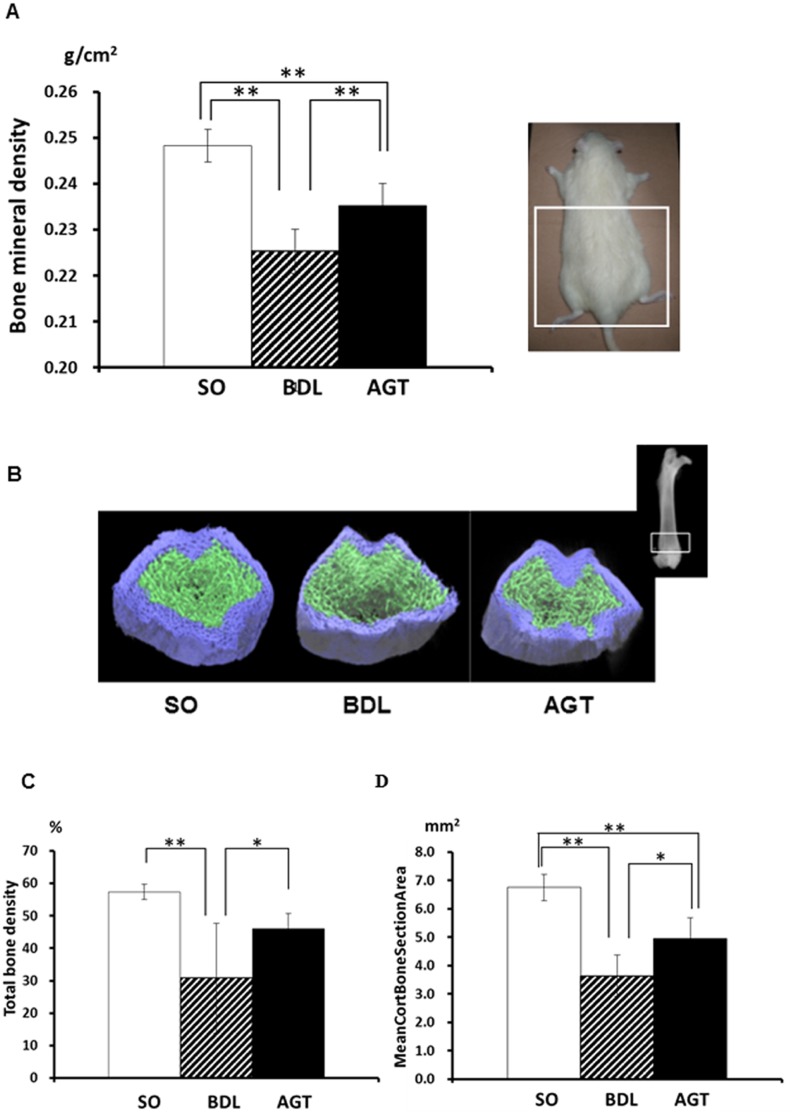
Upregulation of serum γ-glutamyl transpeptidase (GGT) in bile duct ligation animals induces reduction of bone mass. (A) Bone mineral density of rat lower body (white square indicate analyzed area) at 2 w after operation measured by dual-energy X-ray absorptiometry (DEXA). SO, sham operated, control rats; BDL, rats with cholestatic liver disease treated by bile duct ligation; and AGT, BDL rats treated with intraperitoneal injection of GGT antibody (AGT3). (B) Representative μ-CT images of the distal femora of the rats in the SO, BDL, and AGT groups. White squares indicate the area analyzed. (C) Total bone density, (D) Mean cortical bone section area. Significant differences (*P < 0.05, ** *P* < 0.01) were observed between two experimental groups.

Bone mineral density (BMD) of the lower body of BDL rats (0.225±0.005) markedly decreased at 2 weeks after operation (*P* < 0.01) in comparison with that of SO rats (0.248±0.004). In contrast, bone degradation in AGT3-teated rats (AGT) (0.235±0.005) was significantly reduced compared with that in the BDL rats (*P* < 0.01) (Fig 2A).

To confirm the effects of BDL on bone structure, we performed a μ-CT analysis of the distal metaphyseal region of the femora. The total bone mass, in particular the thickness of the cortical bone, decreased in the BDL group. In contrast, prominent improvement of the bone mass was evident in AGT group (Fig 2B). Morphometrical parameters including total bone density (SO; 57.2±2.30, BDL; 30.8±9.75, AGT; 46.1±4.60) (Fig 2C) and mean cortical bone section area (SO;6.7±0.47, BDL; 3.6±0.73, AGT;4.9±0.73) (Fig 2D) also supported the results of these μ-CT images.

### Histomorphometry of bone tissue in bile duct ligation rats

**Fig 3:**
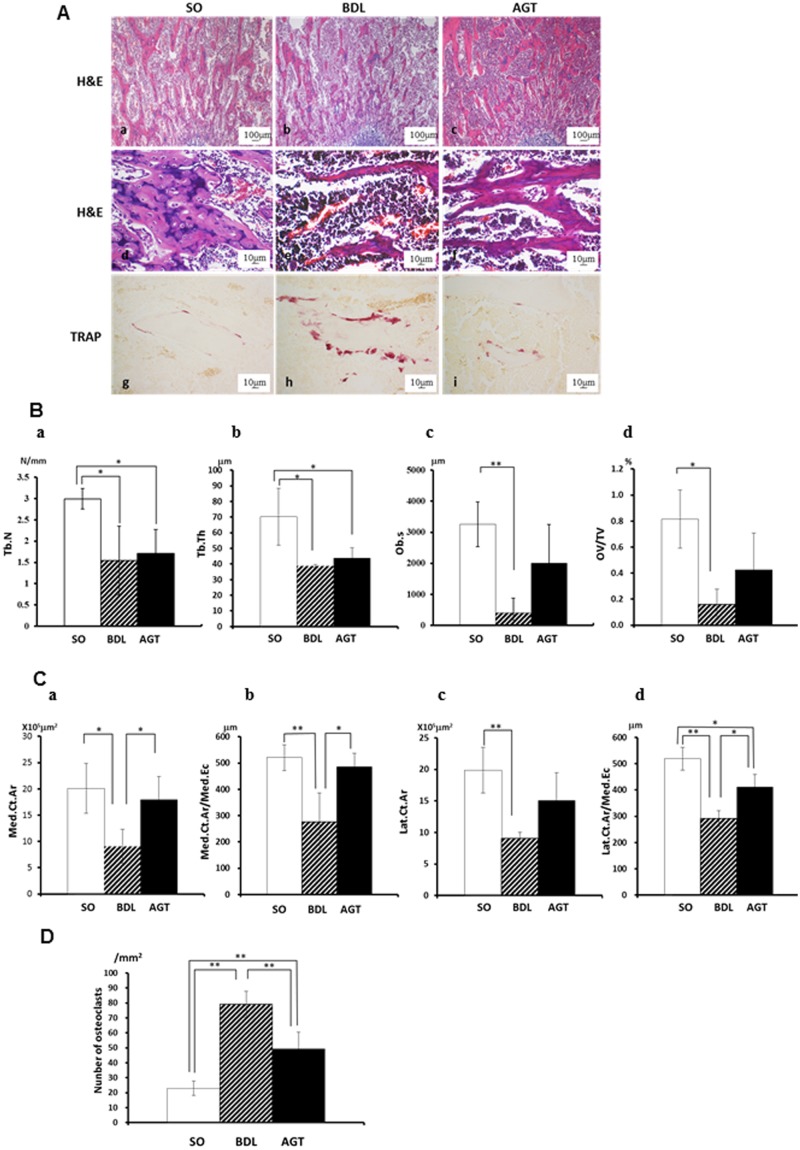
Bile duct ligation animals exhibit upregulated bone resorption with reduced bone formation and AGT3 partially improves them. (A) Histological findings: hematoxylin-eosin (H&E) staining: (a,d) SO, sham operated control rats, (b,e) BDL, rat with cholestatic liver disease treated by bile duct ligation, (c,f) AGT, BDL rats treated with intraperitoneal injection of GGT antibody (AGT3). Tartrate-resistant acid phosphatase (TRAP)-positive osteoclasts: (g) SO rats (h) BDL rats and (i) AGT rats. (B) Histomorphometric analysis of cancerous bone: (a) Trabecular bone number (Tb.N), (b) Trabecular thickness (Tb.Th), (c) osteoblast surface (Ob.S), and (d) osteoid volume per total tissue volume (OV/TV). (C) Histomorphometric analysis of cortical bone: (a) medial cortical area (Med.Ct.Ar), (b) medial cortical area per medial endocortical (Med.Ct.Ar./Med.Ec), (c) lateral cortical area (Lat.Ct.Ar), and (d) lateral cortical area per lateral endocortical (Lat.Ct.Ar./Lat.Ec). (D) Number of TRAP-positive osteoclasts. Significant differences (*P < 0.05, **P <0.01) were observed between the two experimental groups.

Fig 3A shows representative histological findings of the distal edge of the femur from each group. Cancellous bone trabeculae of BDL-treated animals (Fig 3Ab and 3Ae) became thin and less connected and the number of trabeculae decreased compared to that in the SO animals (Fig 3Aa and 3Ad). AGT3 application improved the reduction of bone trabeculae caused by BDL (Fig 3Ac and 3Af). In BDL-treated animals, numerous TRAP-positive–osteoclasts, which were active form, were seen along the surface of the resorbed trabeculae but were fewer and inactive in the SO group (Fig 3Ag and 3Ah). In AGT group, osteoclasts decreased in number and were small and inactive (Fig 3Ai).

Histomorphometry of bone tissues revealed that trabecular number (Tb.N; 1.5±0.8), thickness (Tb.Th; 38.7±0.9), osteoblast surface (Ob.s; 402±272.1) and osteoid volume per total tissue volume (OV/TV; 0.16±0.067) in the BDL group significantly decreased compared to those in the SO group (3.0±0.2, 70.2±18.2, 3255±425 and 0.81±0.22 respectively) (Fig 3B). Although antibody-treatment did not stop trabecular bone degradation (TbN; 1.7±0.6, Tb.Th; 43.8±6.6) in this context (Fig 3Ba and 3Bb), Ob.s (2000±1250) and OV/TV (0.43±0.164) were increased in the AGT group (Fig 3Bc and 3Bd); these results suggest signs of recovery of the trabecular structures. On the other hand, consistent with the results of the μ-CT analysis, we observed significant downregulation of cortical bone structures including medial cortical area (Med.Ct.Ar; 9.1±1.9), medial cortical area per medial endocortical (Med.Ct.Ar./Med.Ec; 276.0±110.3), lateral cortical area (Lat.Ct.Ar; 9.1±0.55), and lateral cortical area per lateral endocortical (Lat.Ct.Ar./Lat.Ec; 291.4±30.3) in BDL group compared to SO group (20.1±4.7, 520.6±49.4, 19.9±3.6 and 518.0±43.5, respectively). They also significantly increased by AGT3-treatment (17.9±4.4, 485.4±51.7, 15.0±4.4 and 411.7±46.7, respectively. *P* < 0.05, Fig 3Ca–3Cd). In addition, the number of TRAP-positive–osteoclasts, which was increased in the BDL group (70.0±8.7), was significantly decreased in the AGT group (49.1±11.3; *P* < 0.01) (Fig 3D). Altogether, our results strongly suggest that significantly elevated GGT level served as a bone-resorbing factor in BDL-induced bone degradation.

### Cytokine array of bone marrow fluid

To determine the changes in the expression levels of bone metabolism-related factors in the bone, we performed a cytokine array analysis using bone marrow supernatant fluid. Table 3 shows the changes in the expression of the 17 cytokines/chemokines evaluated in the bone marrow fluid of the BDL group at 2 weeks after operation. The quantitative analysis of array spots indicated a >1.5-fold increase in the levels of IFN-γ, LIX, MCP-1, TIMP-1, and VEGF-A in the BDL group compared to those in the SO group. These cytokines, except TIMP-1, were well established as osteoclast-forming factors secreted by OBs.

**Table 3:** Cytokine expression in bone marrow at 2 weeks after operation.

Protein Name	Ratio BDL/SO
IFN-γ	3.398029*
LIX	3.220786*
TIMP-1	2.569609*
VEGF-A	1.663466*
MCP-1	1.528099*
CINC2	1.240315
Leptin	1.092309
MIP-3α	1.077039
CX3CL1	1.069715
CINC3	1.065649
TNF-α	1.026223
IL-10	0.988705
IL-1β	0.978116
beta-NGF	0.955919
IL-6	0.93969
IL-1α	0.843519
CSF2	0.833812

* indicates 1.5 fold upregulation

SO: sham-operated group

BDL: bile duct ligated group

### Osteoclastogenic gene expression by γ-glutamyl transpeptidase (GGT)

To validate the upregulation of IFN-γ, LIX, MCP-1, and VEGF-A mRNA expression, RT-PCR was performed using the total RNA extracted from the ST2 cells stimulated by rhGGT. In addition, the changes in expression of other representative bone resorption-related cytokines such as TNF-α, IL-1β, RANKL, and OPG were analyzed.

**Fig 4:**
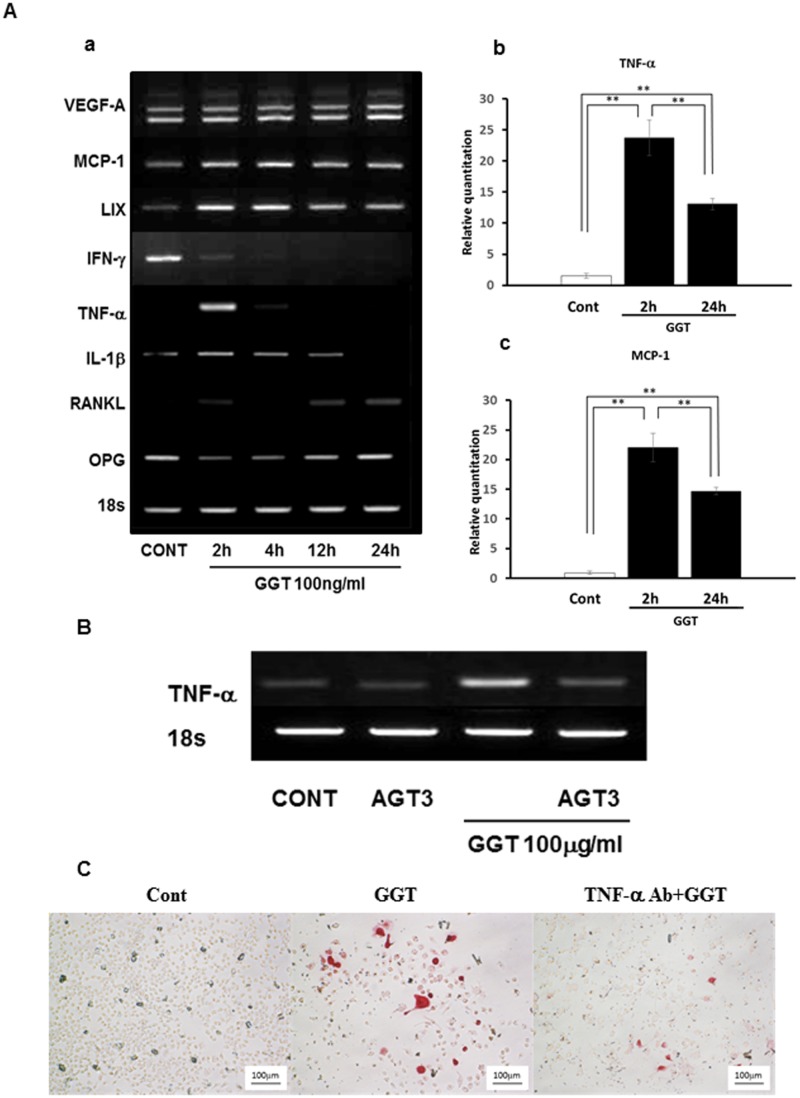
γ-glutamyl transpeptidase (GGT) stimulates osteoclastogenesis-related gene expression in ST2 cells. (A) (a) ST2 cells were treated with 100 ng/ml of recombinant human GGT (rhGGT) for 2, 4, 12 and 24 h. VEGF-A, MCP-1, LIX, IFN-γ, TNF-α, IL-1β, RANKL and OPG mRNA expression was analyzed by RT-PCR. ST2 cells were treated with 100 ng/ml of recombinant human GGT (rhGGT) for 1 and 2d. Quantificaation of TNF-α (b) and MCP-1 (c) was performed by realtime PCR. (B) 100 ng/ml of AGT3 was added 2 h before 100 ng/ml of rhGGT treatment. After 2 h of incubation with rhGGT, TNF-α mRNA expression was examined by RT-PCR. (C) Bone marrow cells were cultured with conditioned medium of GGT-stimulated ST2 with or without TNF-α neutralizing antibody (TNF-α Ab). The cell culture stained with TRAP solution.

Consistent with the results of the cytokine array, VEGF-A, MCP-1, and LIX were upregulated by rhGGT-stimulation in ST2 cells. In addition, TNF-α, IL-1β, and RANKL upregulated by rhGGT. Meanwhile, IFN-γ and OPG mRNA expression were downregulated after rhGGT-stimulation (Fig 4Aa). Quantification of gene expression by real-time PCR showed that GGT induced 23.7-fold upregulation of TNF-α and 22.0-fold upregulation of MCP-1 gene expression at 2 h after GGT stimulation (Fig 4Ab and 4Ac). To further determine the effect of GGT on ST2 cells, we performed an inhibition study on TNF-α mRNA expression, one of the representative molecules induced by GGT stimulation using AGT3. As the result, AGT3 pretreatment significantly reduced TNF-α mRNA expression in GGT-stimulated ST2 (Fig 4B). Indeed, AGT3 reduced pro-IL-1β induced by GGT at protein level. (S1 Fig). Finally, using BMC culture with conditioned medium of GGT stimulated ST2, TRAP-positive preosteoclast and osteoclast formation was observed. In contrast, TNF-α neutralizing antibody partially suppressed GGT-induced osteoclasts (Fig 4C). These results showed that excessive GGT upregulated several bone resorbing factors in ST2 cells, suggesting that GGT might contribute to bone degradation.

### Effects of recombinant human γ-glutamyl transpeptidase (GGT) on osteogenic differentiation

**Fig 5:**
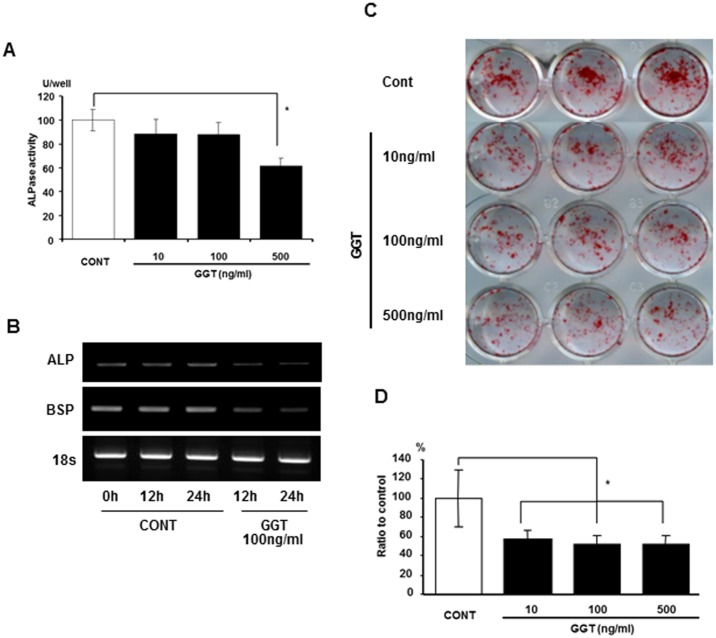
γ-glutamyl transpeptidase (rhGGT) inhibits osteoblastic differentiation of osteoblasts *in vitro*. (A) Alkaline phosphatase (ALP) activity in MC3T3-E1 cells after 6d of incubation with rhGGT (10, 100 and 500 ng/ml). (B) Mineralization-related gene expression in MC3T3-E1 cells at 12 and 24 h after incubation with rhGGT (100 ng/ml). (C) Primary osteoblasts (OBs) were cultured in OB differentiation media for 2 w with rhGGT (10, 100, and 500 ng/ml); Alizarin red S staining is shown for each condition; (D) Quantification of stained Alizarin red.

Since it has been reported that GGT stimulates the activation of stromal cells, we further investigated the influence of GGT on osteogenic differentiation using primary OBs. Primary OBs were cultured in OB differentiation medium with or without various amounts of rhGGT, in order to assess ALP activity. A total of 500 ng/ml of rhGGT significantly downregulated ALP activity in MC3T3-E1 cells (Fig 5A). In addition to ALP mRNA expression, BSP mRNA expression was downregulated 12 h and 24 h after rhGGT stimulation (Fig 5B). After 3 w of culture, rhGGT significantly decreased mineral nodule formation by primary OBs (Fig 5C and 5D; *P* < 0.05). These results indicate that GGT possesses not only osteoclastogenic activity, but also inhibitory activity on bone formation.

## Discussion

Hepatic osteodystrophy is the most common complication in patients with chronic liver disease including cholestatic liver diseases (CLD). Atkinson et al. [14] first reported that 17 of 25 patients with CLD experienced malresorption or diseases of the bone. The prevalence of osteoporosis among patients with advanced chronic liver disease, which has been reported to be between 12% and 55%, was higher than that of primary osteoporosis in the general population.[15] The prevalence is higher in chronic liver diseases, especially in chronic CLDs such as primary biliary cirrhosis and primary sclerosing cholangitis in which high levels of serum GGT are evident.[15] Therefore, in the present study, we used a rat BDL model with bile duct obstruction to examine the role of GGT in the development of hepatic osteodystrophy. The rat BDL model well recapitulates many of the changes described in patients with CLD, such as portal fibrosis and bile duct obstruction with severe portal and lobular inflammation. [16–19] In this model, the serum levels of GGT, well known as the most sensitive marker for hepatic damage by biliary obstruction, [19–21] are upregulated. In addition to liver damage, a decrease in bone mass and reduction of bone formation are evident. [17,18] It is well accepted that the rat BDL model represents the human bone disease in patients with CLD.[17,18,20]

In the present study, activities of serum enzymes, including GGT, AST and ALT, and levels of total bilirubin were significantly elevated, indicating that the BDL group experienced jaundice and liver damage when the bones were examined at 2 w after BDL. Actually, liver injury such as necrosis, fibrosis and bile duct proliferation were observed in the liver of BDL animals. Moreover, the BDL group showed remarkable bone reduction with an increase in the number of osteoclasts. Hiramatsu et al. [10] reported that the GGT transgenic (GGT-Tg) mouse with elevated serum GGT level showed osteopenia with microstructural deterioration. Niida et al. [9] identified GGT as a novel bone-resorbing factor that activated osteoclast formation. The findings reinforced that upregulation of serum GGT level is an important causative factor for bone reduction in patients with CLD.

To clarify the mechanism of osteopenia caused by GGT, we analyzed cytokine/chemokine expression, *in vivo* and *in vitro*. GGT upregulated the expression levels of MCP-1, VEGF-A, LIX, TNF-C, IL-1β, and RANKL mRNA in osteoblastic cells, whereas OPG mRNA expression was downregulated. Moreover, we confirmed that MCP-1, VEGF-A, and LIX mRNA expression was upregulated in bone marrow flushed out of BDL rats. Therefore, it is possible that the excessive GGT-induced osteoclastogenetic molecules in bone marrow stromal cells/osteoblastic cells may contribute to bone reduction in BDL-treated rats. Niida et al. [9] reported that purified GGT protein stimulated osteoclast formation through the expression of RANKL in bone marrow stromal cells independently of its enzymatic activity. In GGT-Tg mice crossed with RANKL-deficient mice, upregulated GGT in serum was not able to induce osteoclastogenesis. This indicates that RANKL is required for GGT action. [10] Moreover, the expression of critical regulators of osteoclastogenesis, i.e., c-Fos, c-Jun, and nuclear factor of activated T cells (NFATc1), were increased in the bone marrow cells of the GGT-Tg model at the molecular level.[10] The finding suggests that GGT may alter the sensitivity of the pre-osteoclast/osteoclast. Furthermore, TNF-α and IL-1β are well accepted to induce osteoclastogenesis in osteoporosis, and inflammatory bone loss associated with rheumatoid arthritis and periodontitis.[21–23] These cytokines are also involved in the modulation of the OPG/RANKL system in patients with chronic liver disease.[21] Especially, TNF-α was known to induce osteoclastogenesis both dependently and independently via RANKL expression in OBs.[24,25] In the present study, a GGT specific antibody, AGT3, significantly suppressed GGT-induced upregulation of TNF-α and IL-1β expression in osteoblastic cells. AGT3 specifically recognizes the large subunit of GGT, but enzymatic activity of GGT exists in the small subunit of GGT.[9] The mutant GGT without enzymatic activity also induced osteoclastogenesis in bone marrow cell culture.[9] These findings indicate that a new epitope showing the cytokine-like activity of GGT exists in the larger subunit. However, a new epitope showing cytokine-like activity and its functional receptor have not been identified to date, and further studies are needed.

The present study showed that GGT significantly inhibited mineral nodule formation and expression of ALP and bone sialo-protein in osteoblastic cells at the molecular level. Using tetracycline-based histomorphometric analysis, Ackerman et al. demonstrated that the bone formation rate in BDL-treated rats decreased.[17] Weineb et al.[18] revealed that bone marrow from BDL-treated rats contains fewer osteoprogenitor cells than that from SO rats. In the GGT-Tg mouse, a lowered bone formation rate with reduced OB surface was also observed.[10] Therefore, direct inhibition of OB proliferation and differentiation by GGT may partially contribute to bone reduction in patients with hepatic osteodystrophy.

Moreover, in the BDL-treated animals, the levels of other molecules including bilirubin and nitric oxide, which inhibit OB proliferation and differentiation, are increased,[16,17] whereas the level of 1,25(OH)_2_D_3_, which decreases bone mineralization, was decreased.[17] In the present study, high levels of total bilirubin and low levels of 1,25(OH)_2_D_3_ were also detected. Although there have been controversial studies in patients regarding the relationship between bilirubin level and hepatic osteodystrophy,[26,27] *in vitro* studies showed that unconjugated bilirubin decreased OB viability, differentiation, and mineralization.[28,29] Vitamin D_3_ deficiency has been widely associated with chronic liver disease.[30] The low level of vitamin D_3_ induces osteomalacia in patients with chronic liver disease.[31] Although the improvement of bone formation by AGT3 application to BDL-treated rats was limited in the present study, it is reasonable to assume that other molecules in the serum were involved in the bone formation rate in cancellous bone.

Since stimulation of osteoclast formation and inhibition of bone formation by GGT may account for the osteopenia induced by chronic hepatic diseases, the blocking of cytokine-like activity of GGT by AGT3 is a potential strategy for the prevention/treatment of hepatic osteodystrophy. Ishizuka et al.[32] reported that intraperitoneal application of AGT3, which specifically reacts with an epitope related to osteoclastogenesis, reduced osteoclast number and bone erosion in CIA mice and suggested that AGT3 might be a novel therapeutic agent for attenuating joint destruction in rheumatoid arthritis patients. In the present study, intraperitoneal injection of AGT3 also significantly improved BDL-induced reduction in BMD, loss of total bone density, and increase in the number of osteoclasts. AGT3, in particular, remarkably improved reduction in cortical bone area and thickness with suppression of osteoclastogenesis. Weinreb et al.[18] also reported that osteopenia in the BDL-treated rats results in reduction of mechanical strength and strength of the tibial diaphysis mainly caused by cortical osteopenia. Therefore, a GGT antagonist such as AGT3, which can improve cortical bone reduction, might be useful as a novel therapeutic agent for hepatic osteodystrophy and may prevent traumatic fractures in patients with chronic liver disease.

Interestingly, BDL-induced liver injury was improved in AGT3 applied group, indicating inhibition of cytokine-like activity of GGT by AGT3. This inhibitory effect might bring beneficial impacts on liver injury. There is possibility that the improvement of liver injury by AGT3 application may indirectly lead to inhibition of bone reduction in addition to direct inhibition of osteoclastogenesis by GGT *in vivo* AGT rats. The control of upregulated serum GGT in patients with CLD may be critical for not only preventing hepatic osteodystrophy but also improving liver injury.

As described above, the GGT-Tg mouse with approximately 200 IU/L GGT activity in the serum showed osteoporosis.[10] This indicates the possibility that even moderately elevated circulating levels of GGT associated with CLD and biliary tract disease would contribute to secondary bone loss in patients. A recent review by Targher [33] clarified evidence for the association of high normal serum GGT enzyme activity, mostly within the reference range, with the risk of mortality and major vascular (i.e., cardiovascular morbidity and mortality) and non-vascular outcomes (i.e., incident type 2 diabetes, chronic kidney disease, and cancer). Although GGT was generally considered to be a biomarker for oxidative stress associated with glutathione regulation and degradation, the cytokine-like activity of GGT, demonstrated in this study, might be responsible for not only hepatic osteodystrophy but also the establishment of other diseases. Further additional research will be needed to evaluate the important roles of GGT in pathogenesis.

In conclusion, the findings herein indicate that elevated serum GGT level reduces bone mass by suppression of bone formation and promotion of osteoclastogenesis via OBs and suggest that upregulation of GGT in the serum is a potent risk factor for hepatic osteodystrophy. Moreover, AGT3, which can inhibit cytokine-like activity of GGT, may be a novel preventive/therapeutic agent for secondary osteoporosis in patients with chronic liver disease.

## Supporting Information

S1 Fig AGT3 inhibits γglutamyl transpeptidase (GGT) induced pro IL-1β protein production in ST2 cells. 100 ng/ml of AGT3 was added 2 h before 100 ng/ml of rhGGT treatment. After 2 h of incubation with rhGGT, pro IL-1β protein expression was examined by western blot.
(TIF)

S1 Table Biochemical parameters. Seum levels of biochemical markers at 2w after BDL operation. SO, sham-operated group; BDL, bile duct ligated group.
(DOCX)
