# Supplementary figures and images for: Osteodystrophy in Cholestatic Liver Diseases Is Attenuated by Anti-γ-Glutamyl Transpeptidase Antibody
Source: PLoS One. 2015 Sep 29;10(9):e0139620. doi: 10.1371/journal.pone.0139620 (PMC4587927; doi:10.1371/journal.pone.0139620)

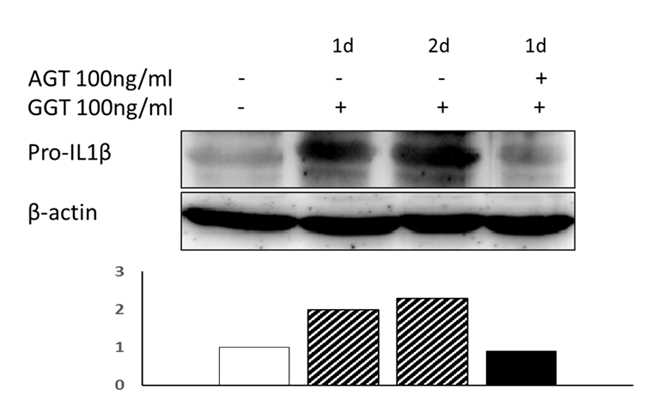

Supplement: S1 Fig — 100 ng/ml of AGT3 was added 2 h before 100 ng/ml of rhGGT treatment. After 2 h of incubation with rhGGT, pro IL-1β protein expression was examined by western blot. (TIF) [file pone.0139620.s001.tif]
