# Supplementary material for: Osteodystrophy in Cholestatic Liver Diseases Is Attenuated by Anti-γ-Glutamyl Transpeptidase Antibody
Source: PLoS One. 2015 Sep 29;10(9):e0139620. doi: 10.1371/journal.pone.0139620 (PMC4587927; doi:10.1371/journal.pone.0139620)
Supplement: S1 Table — Seum levels of biochemical markers at 2w after BDL operation. SO, sham-operated group; BDL, bile duct ligated group. (DOCX) [file pone.0139620.s002.docx]

| S1 Table. |  |  |  |  |  |  |  |
| --- | --- | --- | --- | --- | --- | --- | --- |
| Biochemical parameters |  |  |  |  |  |  |  |
|  |  |  |  |  |  |  |  |
|  | **SO** | | | **BDL** | | | **P-value** |
| Total bilirubin(mg/dl) | 0.00 | ± | 0.00 | 8.14 | ± | 0.69 | < 0.0001 |
| 1,25(OH)_2_D_3_ (pg/ml) | 265.70 | ± | 39.08 | 158.30 | ± | 30.00 | < 0.0001 |
|  |  |  |  |  |  |  |  |
| SO, sham-operated group; BDL, bile duct ligated group; | | | |  |  |  |  |
